# A Case Report of Mesenteric Panniculitis

**DOI:** 10.7759/cureus.6764

**Published:** 2020-01-24

**Authors:** Parth Mehta, Anil Kumar Reddy Reddivari, Mansoor Ahmad

**Affiliations:** 1 Internal Medicine, University of Illinois College of Medicine, Peoria, USA

**Keywords:** mesenteric panniculitis, pancreatitis, abdominal pain, alcohol abuse

## Abstract

A 33-year-old male with a past medical history of hypertension, migraine, depression, and alcohol abuse presented with complaints of nausea, vomiting, and abdominal pain. He was found to have an elevated lipase and ethanol level. CT scan of the abdomen was done which showed peripancreatic fat stranding around the pancreatic head consistent with mild acute uncomplicated pancreatitis. CT scan of the abdomen also revealed multiple clustered nodes in the central mesentery with fatty haziness and ground-glass appearance of the mesenteric fat, representing mesenteric panniculitis.

## Introduction

Mesenteric panniculitis (MP) is a fibrosing inflammatory disease affecting the adipose tissue of the mesentery. It can be acute or chronic and is associated with abdominal trauma, abdominal surgery, malignancies, inflammatory, granulomatous, and autoimmune conditions. It was first described in 1924 by Jura and is also known as sclerosing mesenteritis, lipsclerotic mesenteritis, mesenteric lipodystrophy, or mesenteric Weber-Christian disease. It is most commonly diagnosed incidentally during an imaging study, mainly a CT scan, which is the most sensitive imaging test but a definitive diagnosis requires biopsy. It can be asymptomatic but can also present with abdominal pain, nausea, vomiting, diarrhea or constipation, intestinal obstruction, or a palpable abdominal mass. Laboratory results are usually normal in the absence of other conditions. It is a self-limiting disease and may show signs of improvement without treatment on follow-up imaging studies. Sometimes, symptoms can improve but the imaging findings do not change if there is a persistent underlying cause. Prognosis is good due to its benign nature [1]. We report a case of mesenteric panniculitis in a young male.

## Case presentation

A 33-year-old male with a past medical history of hypertension, depression, and alcohol abuse presented with complaints of nausea, vomiting, and abdominal pain. He had a history of tobacco and alcohol abuse. He was drinking six cans of beer and a half-pint of vodka daily for the last six months. He did not have any significant medical issues in his family and family history was negative for any cancers. His temperature was 97.8° F, blood pressure was 136/88 mmHg, pulse was 87/minute, and respiratory rate was 14/minute. Physical examination was positive for moderate tenderness in the epigastrium on deep palpation.

Initial workup revealed laboratory values as mentioned in Table 1.

**Table 1:** Initial lab values upon presentation AST: Aspartate aminotransferase, ALT: Alanine aminotransferase

Test	Results	Reference value
Hemoglobin (g/dl)	14	13-17
Hematocrit (%)	39.8	39-49
White blood cells (10*3/uL)	6.1	3.60-9.50
Platelets (10*3/uL)	178	150-440
AST (U/L)	124	10-50
ALT (U/L)	71	6-40
Lipase (U/L)	2634	70-400
Ethanol (mg/dl)	152	0-9

**Figure 1:**
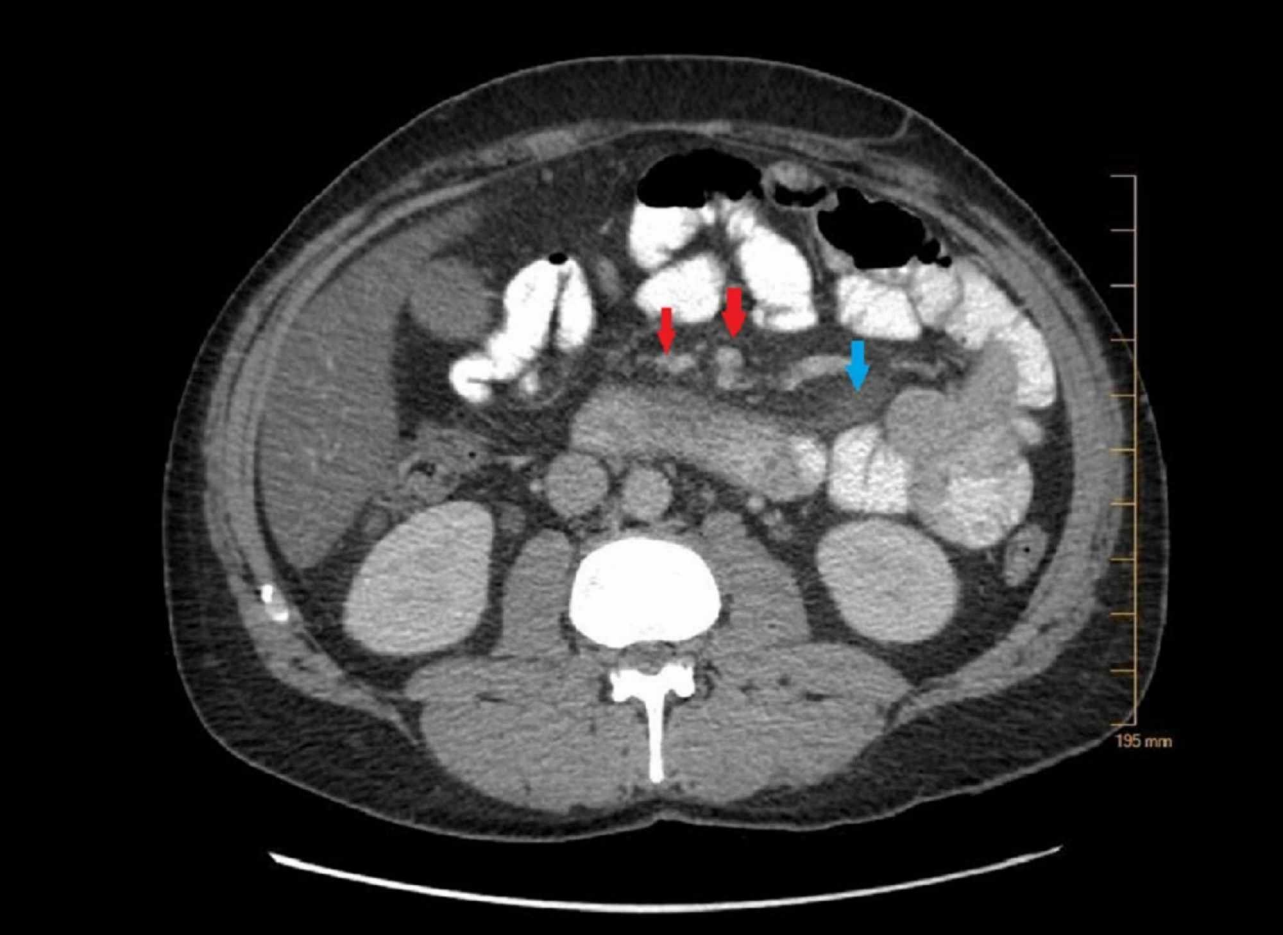
Image from CT scan of the abdomen performed with oral and intravenous contrast The red arrows show multiple clustered nodes in the central mesentery with fatty haziness, which is also known as "misty mesentery," and the blue arrow shows the ground-glass appearance of the mesenteric fat representing mesenteric panniculitis.

CT scan of the abdomen was done, which showed peripancreatic fat stranding around the pancreatic head consistent with mild acute uncomplicated pancreatitis. CT scan of the abdomen also revealed multiple clustered nodes in the central mesentery with fatty haziness which is also known as "misty mesentery" and ground-glass appearance of the mesenteric fat representing mesenteric panniculitis (Figure 1).

**Figure 2:**
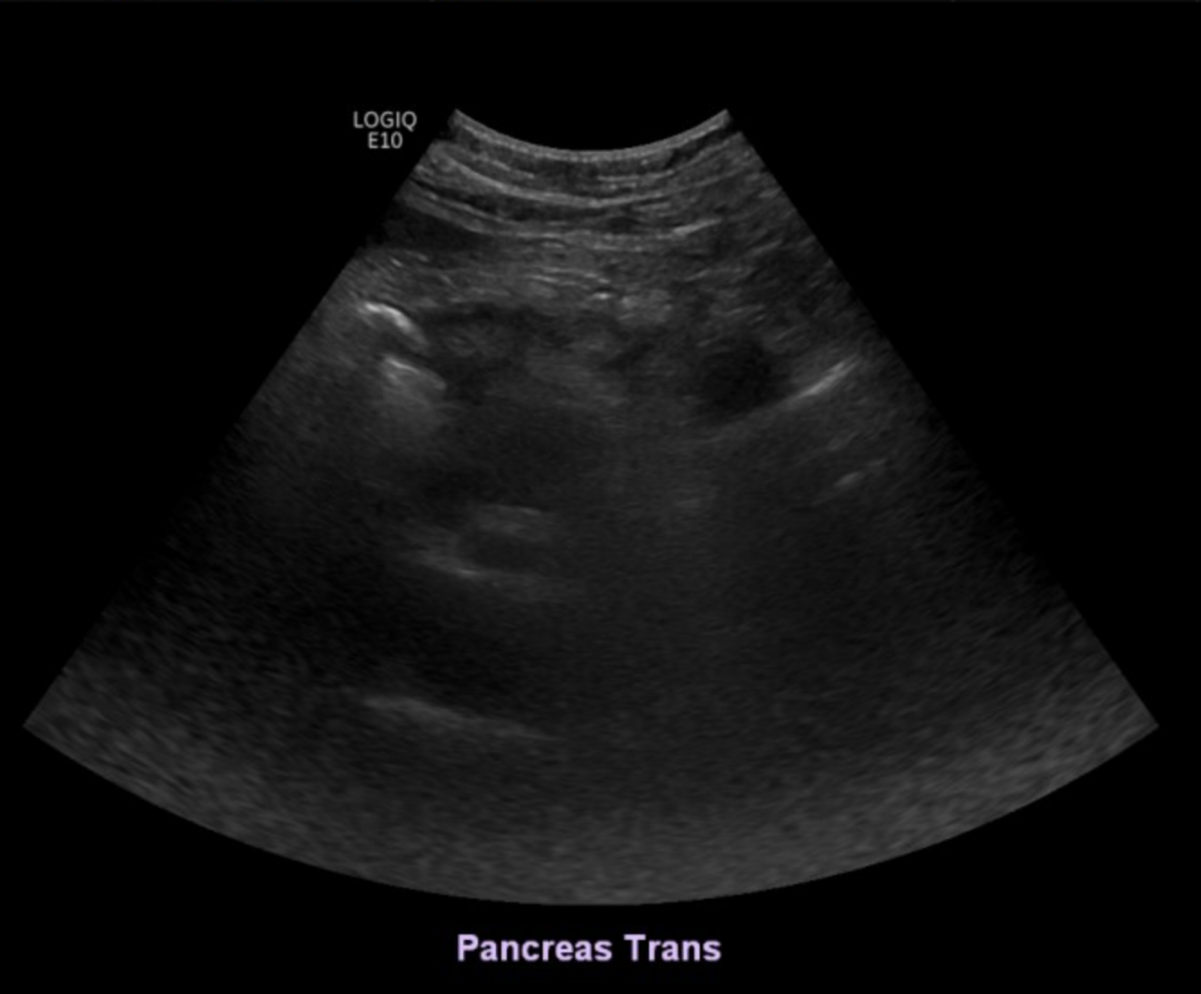
The pancreas was obscured by bowel gas and was not visualized well on the ultrasound

The pancreas was obscured by bowel gas and was not visualized well (Figure 2).

The patient was started initially on conservative management for suspected acute pancreatitis. He was kept nothing-by-mouth and was started on intravenous fluids and intravenous pain medications. On the second day after admission, all laboratory abnormalities were improving. AST and ALT levels had normalized. The lipase level was down to 926 U/L. The patient was still having abdominal pain, but it was now around the umbilicus and not in the epigastrium. Nausea and vomiting remained persistent. On the third day, his lipase level came down to 267 U/L and had normalized. Complete blood count, metabolic panel, and hepatic function remained unremarkable. Lactic acid was checked and returned normal.

**Figure 3:**
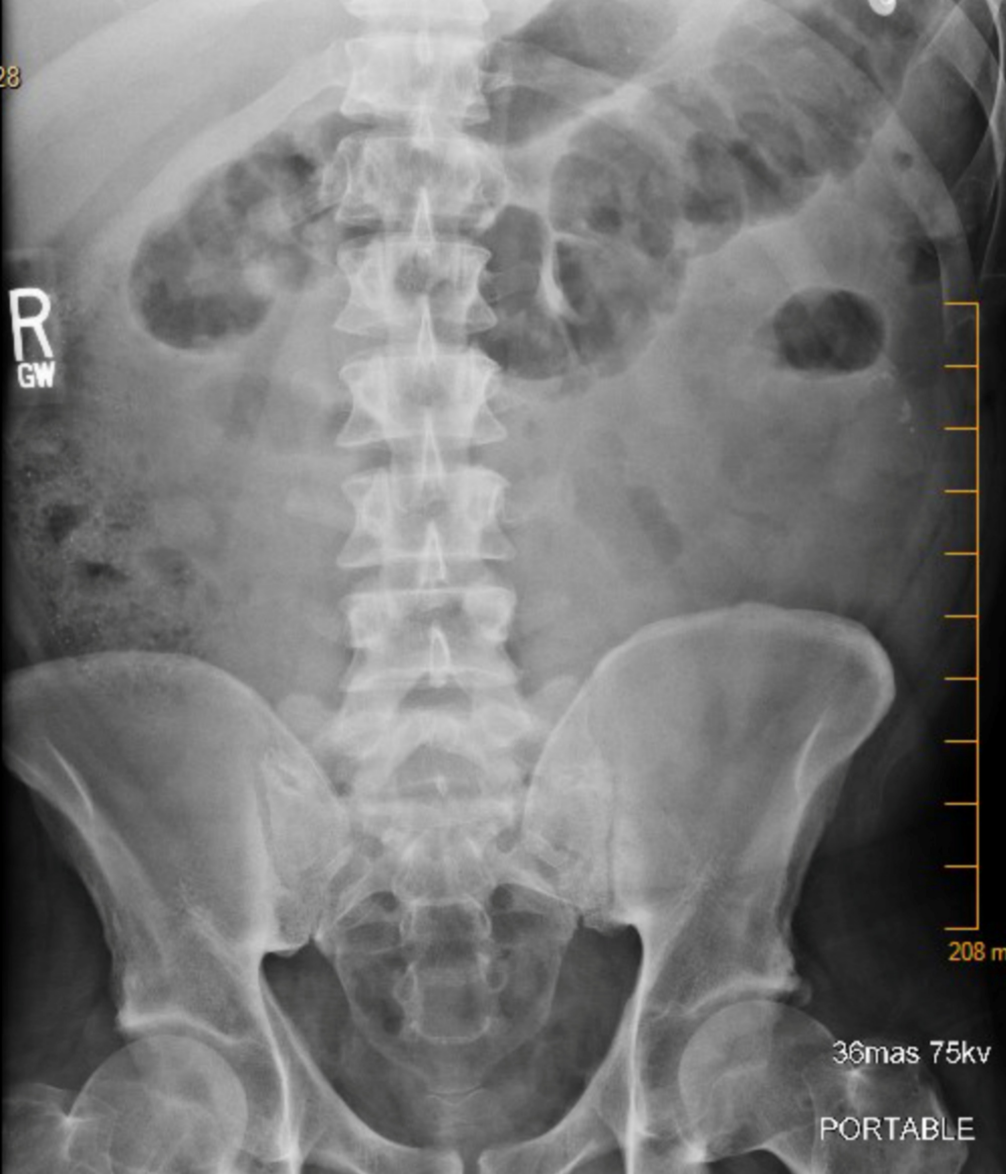
An x-ray of the abdomen showed a small amount of stool in the ascending colon but was negative for small bowel obstruction

An abdominal X-ray was done and came back negative for small bowel obstruction and showed a small amount of stool in the ascending colon (Figure 3).

Due to persistent abdominal pain, nausea, and vomiting, a gastroenterology consultation was obtained. His symptoms were believed to be secondary to mesenteric panniculitis seen on the CT scan and not secondary to acute pancreatitis. The patient was started on 40 mg of oral prednisone daily. After starting prednisone, his symptoms completely resolved within 36 hours. He started tolerating diet and was discharged home with a gastroenterology follow-up. He was prescribed prednisone oral taper over a period of two weeks.

**Figure 4:**
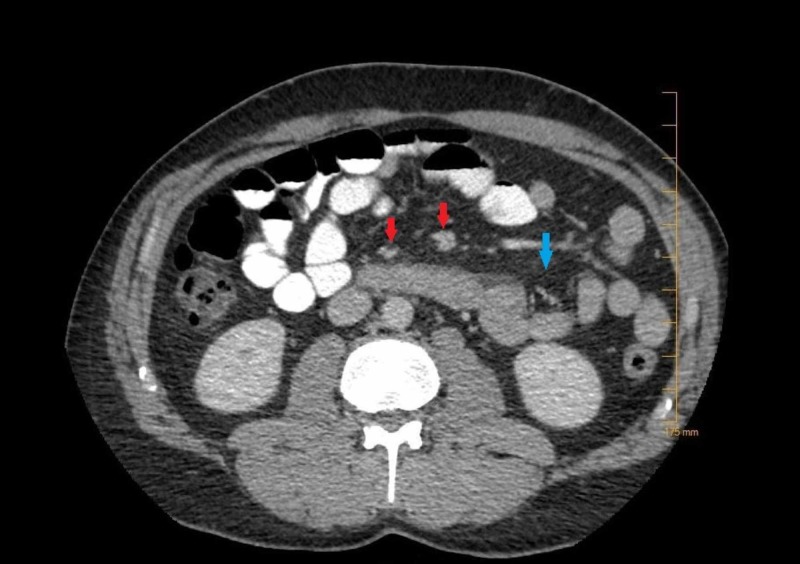
Image from the follow-up CT scan of the abdomen performed with oral and intravenous contrast The red arrows show persistent multiple clustered nodes in the central mesentery with fatty haziness and the blue arrow shows resolution of the ground-glass appearance of the mesenteric fat.

The patient was closely followed after discharge and had a repeat CT scan of the abdomen done at six months, which showed the complete resolution of the ground-glass appearance of the fat but the adenopathy persisted (Figure 4).

The patient was completely asymptomatic by this time but had unfortunately continued to drink alcohol although he had cut down his frequency of drinking to three to four days in a week but had not cut down on the amount of alcohol. On a repeat CT scan of the abdomen, the patient was again found to have findings of mesenteric panniculitis with persistent adenopathy. He was eventually released from gastroenterology care.

## Discussion

MP is a rare fibrosing inflammatory disease characterized by acute or chronic inflammation affecting, mainly, the small and, less commonly, the large intestinal mesenteric adipose tissue. It can be both acute and chronic and is more commonly found in men as compared to women with a male-to-female ratio of 2-3:1; it is more common in Caucasian men [2].

The exact mechanism of pathogenesis is not clear but the non-specific immune response to various stimuli has been suggested to trigger the inflammation of the adipose tissue. It can be associated with inflammatory conditions such as gastritis and pancreatitis, malignancies such as mesenteric tumors or non-Hodgkin's lymphoma, and granulomatous, rheumatic, and autoimmune diseases such as primary sclerosing cholangitis. It has also been reported to be associated with peptic ulcer disease, thermal or chemical injuries, gall stones, abdominal trauma, abdominal surgery, and infections such as typhoid, tuberculosis, and so on. Mesenteric panniculitis findings have also been seen on imaging in patients with mesenteric ischemia or small bowel obstruction [3]. Patients can have a variety of presentations from being asymptomatic to having abdominal pain, nausea, vomiting, diarrhea or constipation, intestinal obstruction, or a palpable abdominal mass, and symptoms can last from a few days to few years. Occasionally patients can have malaise and weight loss also [4].

Occasionally, anemia and hypoalbuminemia can be seen but labs can be normal in many patients except inflammatory markers, including the erythrocyte sedimentation rate, and C-reactive protein may be elevated in the majority of patients due to the inflammatory nature of the disease [5-6]. Mesenteric panniculitis is usually discovered incidentally on imaging studies for the evaluation of abdominal symptoms. The CT scan is the imaging test of choice and is considered the most sensitive test but cannot be used to confirm the diagnosis and a biopsy may be required to confirm the diagnosis [7]. The CT scan findings can vary based on the severity of inflammation and fibrosis but, in most cases, findings of a fat ring (halo) sign, ground-glass appearance of the mesenteric fat, fatty haziness (misty mesentery), fibrotic or inflammatory soft tissue mass with adenopathy occasionally encasing vessels with thrombosis, calcifications due to adipose tissue necrosis, pseudocapsule, and so on can be seen [8].

Confirmation with pathology may be required with CT scan findings of the soft tissue mass or suspicious adenopathy to rule out other etiologies such as malignancies or granulomatous diseases but it is not recommended. If the follow-up imaging studies show resolution of the soft tissue mass or adenopathy, the biopsy may not be necessary. Since biopsy is invasive, the risks versus the benefits of biopsy should be considered on a case-by-case basis. When the biopsy is obtained, the most common histologic findings are chronic inflammation and fibrosis [9].

The differential diagnosis of mesenteric panniculitis includes disease that can affect mesentery such as non-Hodgkin's lymphoma, peritoneal carcinomatosis, involving mesentery, tumors arising primarily in the mesentery, carcinoid tumor, mesenteric edema caused by conditions such as congestive heart failure, liver cirrhosis, abdominal trauma, and so on [10]. It is important to keep in mind other conditions that can mimic the symptoms and CT scan signs of mesenteric panniculitis and if suspected, a thorough workup should be done.

The main goal of treatment is the relief of symptoms. If patients are asymptomatic, no treatment is required and most patients remain asymptomatic during follow-up also. Treatment does not always prevent complications, but it improves the symptoms in the majority of patients. If the patient is presenting with symptoms of bowel obstruction or mesenteric ischemia due to thrombosis of the vessel, treatment should be offered. Treatment can be offered with steroids, tamoxifen, azathioprine, cyclophosphamide, colchicine, and, sometimes, radiotherapy. The majority of patients who have a higher degree of inflammation respond very well to steroids and tamoxifen. The patients who don't respond to tamoxifen can be treated with azathioprine. In some cases, surgery is required when patients develop obstructive or compressive symptoms. A follow-up CT scan is not always necessary but can be done; however, imaging findings may or may not show resolution [11].

## Conclusions

Mesenteric panniculitis is a rare condition. In asymptomatic patients, it is usually found incidentally on imaging studies during the workup for non-specific abdominal symptoms but even in patients who are symptomatic, diagnosis can be difficult, as many other conditions can also have similar symptoms. The CT scan is the most sensitive test and but cannot be used for confirming the diagnosis. The purpose of this case was to create more awareness about the condition, as improved understanding can help in the timely and better recognition of this condition and can help develop less invasive diagnostic methods and improve its treatment to prevent future complications.

## References

[1] Kaya C, Bozkurt E, Yazıcı P, İdiz UO, Tanal M, Mihmanlı M (2018). Approach to the diagnosis and treatment of mesenteric panniculitis from the surgical point of view. Turk J Surg.

[2] Issa I, Baydoun H (2009). Mesenteric panniculitis: various presentations and treatment regimens. World J Gastroenterol.

[3] Patel N, Saleeb SF, Teplick SK (1999). General case of the day. Mesenteric panniculitis with extensive inflammatory involvement of the peritoneum and intraperitoneal structures. Radiographics.

[4] Akram S, Pardi D, Schaffner J (2007). Sclerosing mesenteritis: clinical features, treatment, and outcome in ninety-two patients. Clin Gastroenterol Hepatol.

[5] Sharma P, Yadav S, Needham CM, Feuerstadt P (2017). Sclerosing mesenteritis: a systematic review of 192 cases. Clin J Gastroenterol.

[6] Ginsburg PM, Ehrenpreis ED (2002). A pilot study of thalidomide for patients with symptomatic mesenteric panniculitis. Aliment Pharmacol Ther.

[7] Daskalogiannaki M, Voloudaki A, Prassopoulos P, Magkanas E, Stefanaki K, Apostolaki E, Gourtsoyiannis N (2000). CT evaluation of mesenteric panniculitis: prevalence and associated diseases. AJR Am J Roentgenol.

[8] van Putte-Katier N, van Bommel EF, Elgersma OE, Hendriksz TR (2014). Mesenteric panniculitis: prevalence, clinicoradiological presentation and 5-year follow-up. Br J Radiol.

[9] Emory TS, Monihan JM, Carr NJ, Sobin L (1997). Sclerosing mesenteritis, mesenteric panniculitis and mesenteric lipodystrophy: a single entity?. Am J Surg Pathol.

[10] Sheth S, Horton KM, Garland MR, Fishman EK (2003). Mesenteric neoplasms: CT appearances of primary and secondary tumors and differential diagnosis. Radiographics.

[11] Nyberg L, Björk J, Björkdahl P, Ekberg O, Sjöberg K, Vigren L (2017). Sclerosing mesenteritis and mesenteric panniculitis - clinical experience and radiological features. BMC Gastroenterol.

